# Physical simulation models for veterinary surgical training: a systematic review

**DOI:** 10.3389/fvets.2026.1880121

**Published:** 2026-08-19

**Authors:** Javier Salas-Guerra, Juan A. Sánchez-Margallo, David Durán-Rey, Francisco M. Sánchez-Margallo

**Affiliations:** 1 Laparoscopy Unit, Jesús Usón Minimally Invasive Surgery Centre, Cáceres, Spain; 2 Robotics, Image-Guided Surgery and Bioprinting Unit, Jesús Usón Minimally Invasive Surgery Centre, Cáceres, Spain; 3 Scientific Direction, Jesús Usón Minimally Invasive Surgery Centre, Cáceres, Spain

**Keywords:** education, surgical simulation, training models, validation, veterinary surgery

## Abstract

**Introduction:**

Simulation has become an increasingly important educational strategy in veterinary surgical training. This is driven by ethical considerations, the need to standardize skill acquisition, and limitations associated with training based on live animals and cadavers. However, the characteristics, scope, and validation of simulators used in veterinary surgery remain heterogeneous and insufficiently systematized. This study aimed to systematically review and characterize the simulators used in veterinary surgical education, focusing on their characteristics, applications, and validations. This should also identify current limitations and potential future research directions.

**Methods:**

A systematic review was conducted following the PRISMA 2020 guidelines. The PubMed and Web of Science databases were searched to identify studies on the use of physical simulators in veterinary surgical training published between July 2016 and July 2026. Studies based solely on cadavers, ex vivo models, virtual reality simulators, and those not focused on veterinary practice, were excluded. The results were synthesized descriptively using predefined categorical frameworks.

**Results:**

A total of 1,409 articles were identified, of which 78 studies were ultimately included. Most simulators were low fidelity (55%) and developed in academic settings. High-fidelity simulators were predominantly commercial (79%). The majority of the simulators identified were canine models (42%) or box trainers (21%), with a focus on sterilization procedures. Comparatively limited representation was found for other species and surgical specialties. Validation approaches were heterogeneous, with face, content and construct validity being the most frequently reported.

**Conclusion:**

Physical simulators are widely used in veterinary surgical education, but their development remains uneven across species, specialties, and validation standards. There is a clear need for higher-fidelity, species-diverse simulators, as well as more rigorous and standardized validation frameworks to strengthen the educational and clinical impact of simulation-based training.

## 1 Introduction

Surgery is a fundamental discipline within veterinary medicine and a core component of clinical practice encompassing procedures that range from basic techniques to complex interventions. During undergraduate training, students are required to acquire a set of essential skills, including the appropriate handling of surgical instruments, an understanding of the principles of surgical asepsis, and appropriate wound management (1).

Traditional surgical education in veterinary schools typically begins with foundational theoretical components, followed by clinical sessions and practical training in skills laboratories, and ultimately progresses to assisted participation in real clinical cases (2). These approaches combine theory and clinical reasoning with mentored hands-on practice, fostering progressive learning in accordance with the principles of “see one, do one, teach one” (3).

Currently, residency programs constitute the cornerstone of veterinary specialization, providing structured training that combines supervised clinical practice with feedback from experienced professionals (4). Although training remains primarily centered on real clinical cases, complementary resources, such as ex vivo models, are also widely used to support technical skill development (4).

In this context, simulation has emerged as an alternative to the use of live animals, providing a controlled and adaptable learning environment that promotes active engagement and allows training to be tailored to individual learner needs (5). Among the alternatives described in the scientific literature are cadaveric models, which offer anatomical fidelity but are limited by postmortem tissue changes and the absence of physiological responses such as bleeding (6). Although cadaver use has historically been widespread in both human and veterinary medicine, current trends indicate a decline and progressive replacement by other modalities (7, 8). Virtual reality and augmented reality simulators represent emerging technologies that enhance realism and immersion, in some cases incorporating haptic feedback (9–11). Additionally, physical artificial simulators encompass a wide range of realism levels and materials, from low-cost academic models to high-fidelity industrially developed simulators, enabling repetition and refinement of basic surgical skills and specific surgical techniques (12).

Simulators have become a fundamental resource in the education of veterinary students and residents (4, 13). However, there is currently limited homogeneity in the surgical specialties and animal species in which they are applied (14, 15), as well as a lack of standardization in the validation processes for new developments within academic fields (14).

In other health science-related disciplines, such as human medicine, simulation-based training is also widely used in both undergraduate and graduate education. In the field of human surgery, Shahrezaei et al. (16) described the use of artificial physical simulators and cadaver models as training modalities that improve surgical education and skill acquisition, in line with trends observed in the veterinary field. Other studies in the health sciences emphasize the need for more methodologically robust, technique-oriented simulation research with more transparent reporting, enabling meaningful comparisons between training modalities and strengthening the empirical basis supporting their educational and clinical impact (17–19).

Furthermore, the use of experimental animal models in surgical training provides an environment capable of reproducing complex clinical scenarios with high realism, such as unexpected hemorrhage or anatomical variability amongst patients, allowing exposure to complications that are difficult to replicate using artificial simulators (20). Nevertheless, experimental model use has been the subject of ethical debate, and current trends align with the Three Rs principle, which promotes the replacement, reduction, and refinement of animal models in experimentation (21, 22). In light of these limitations, there seems to be a need to develop new educational strategies that provide adequate surgical training while safeguarding animal welfare.

Accordingly, this study aimed to systematically review and characterize the simulators used in veterinary surgical education, evaluating its current state of research and to identify existing knowledge gaps and future research directions.

## 2 Methods

We conducted a systematic literature review to assess the current state of simulation models for veterinary surgical training following the Preferred Reporting Items for Systematic Reviews and Meta-Analyses (PRISMA) guidelines (23).

### 2.1 Search criteria

A structured and comprehensive literature search strategy was implemented utilizing PubMed and Web of Science scientific databases. This search included articles from July 2016 to July 2026, to identify recent developments and current practices in this context. A set of keywords pertaining to simulators in veterinary surgery was assessed to ensure the inclusion of pertinent studies. The search string employed in this systematic review was “((train*) OR (teach*) OR (educat*) OR (clinical) OR (skill*))AND (veterinar* OR vet) AND (simulat*) AND (surg*)” in PubMed, and “ALL = (((train*) OR (teach*) OR (educat*) OR (clinical) OR (skill*))AND (veterinary OR vet) AND (simulat*) AND (surg*))” in Web of Science.

### 2.2 Selection of articles

The articles selected for this review were predetermined based on inclusion and exclusion criteria to ensure alignment with its objectives. For the comprehensive analysis of each study included in this review, the Parsifal web tool v2.2 (24) was employed for the systematic search and analysis of scientific articles.

The articles were limited to those that focused on veterinary surgery simulators, published between July 2016 and July 2026. These articles were required to have full-text accessible, either through open-access sources, the authors’ institutional subscriptions or by purchasing it, and to report on the application of these simulators in surgical training contexts.

The exclusion criteria encompassed studies that did not utilize surgical simulators, those that did not incorporate a physical, hands-on model, studies solely based on cadaveric or ex vivo specimens, those focused on human medicine, articles unrelated to the topic, as well as reviews, letters to the editor, book chapters, and conference proceedings.

### 2.3 Data collection and analysis

For each article included in this systematic review, the following parameters were analyzed: species represented, anatomical region, surgical approach, task or procedure performed, fidelity level, scope of application, simulator type, participant profile, evaluation method and type of validation. Articles including more than one simulator were analyzed individually.

Types of simulators: We distinguish between commercial simulators, which are distributed by companies, and academic simulators. The latter are typically developed within the context of research, teaching initiatives, or through manual construction, manufactured with readily available and cost-effective materials.

Scope of application: Type of surgical intervention or procedure for which the simulator is designed. Basic surgical skills training, it includes acquisition of basic surgical skills prior to performing complex procedures; Soft tissue intervention, it includes surgical tasks and procedures related to soft tissues (non-bony structures); Interventional radiology, which includes minimally invasive image-guided procedures involving vascular access; Orthopedics and traumatology, including surgical tasks involving bones, joints or musculoskeletal trauma; Neurosurgery, which are surgical procedures and tasks affecting the central or peripheral nervous system, such as interventions on the brain or spinal cord; Diagnostic procedures, these are techniques aimed at obtaining clinical information or confirming a diagnosis, such as image-guided punctures or orthopedics assessments.

Approach: This refers to the set of procedures performed during surgery to access the anatomical regions. Types of approaches include open surgery, endoscopic, laparoscopic, image-guided surgery and robot assisted.

Tasks: The complexity of tasks that can be performed with the simulator. These include basic tasks, which serve as training exercises for fundamental skills such as cutting, instrument handling, and suturing, and advanced tasks that are designed to train complete surgical procedures.

Fidelity: Given the absence of universally accepted framework for defining simulator fidelity, a three-level classification was developed based on previously published studies (25, 26). This feature aims to classify simulators according to their degree of realism, such as visual appearance, feel, tissue behavior, or response to instruments. Thus, low-fidelity simulators enable the practice of basic skills, with simplified anatomical appearance and functionality. Medium-fidelity systems simulate anatomical structures in a partial or simplified way with a certain degree of tactile or visual realism, including anatomical models or structures with a moderate degree of accuracy. High-fidelity simulators reproduce the anatomy, texture, and response of tissues in detail and with a high degree of realism and may include physiological responses or organs that resemble reality.

Validation: The simulators analyzed were classified based on the type of validation they had successfully achieved. Among the broadly defined types of validation (12, 27–29), for this review we consider face validity, which assesses the visual and functional similarity between the simulator and the real surgical activity based on the subjective opinion of users; content validity, which determines the degree to which the simulator covers the relevant aspects of the technique to be trained according to expert criteria; construct validity, which determines whether the simulator is capable of differentiating between different levels of user skill or experience; predictive validity, which assesses whether the simulator can predict surgical performance in real situations; and concurrent validity, which compares the performance of the simulator with another standard surgical assessment method that measures the same surgical skills or parameters.

Data extraction was performed by a single reviewer. To minimize errors, a subsequent cross-check was performed on the extracted data. Results from the review synthesis will be presented in the result section in the form of tables, graphs and/or percentages.

### 2.4 Risk of bias assessment

The methodological quality of the included studies was examined through an assessment of risk of bias. Although broader critical appraisal tools are available, as Joanna Briggs Institute or MMAT (30, 31), the present review focused specifically on potential threats to internal validity that could affect the reliability of the reported findings by Cochrane methodology (32). Therefore, to assess the bias of the included studies, we used version 2 of the Cochrane Risk of Bias assessment (RoB 2.0) and the Risk of Bias in Non-Randomized Intervention Studies (ROBINS-I) tools, both validated and published. For randomized studies, RoB 2.0 tool was used (33). This tool assesses six criteria: the randomization process, deviations from planned interventions, outcome measurement, missing outcome data, selection of reported outcomes, and overall bias. Each criterion is classified into one of three categories: high risk, some concern, and low risk. For non-randomized studies, the ROBINS-I tool was used (34). This tool assesses several areas, including pre-intervention, confounding factors, participant selection, classification of interventions, deviations from planned interventions, missing data, and outcome measurement. Risk of bias is classified as low, moderate, serious and critical. In case of discrepancies in the assessment of the criteria, an external expert assessor was consulted.

## 3 Results

**Figure 1:**
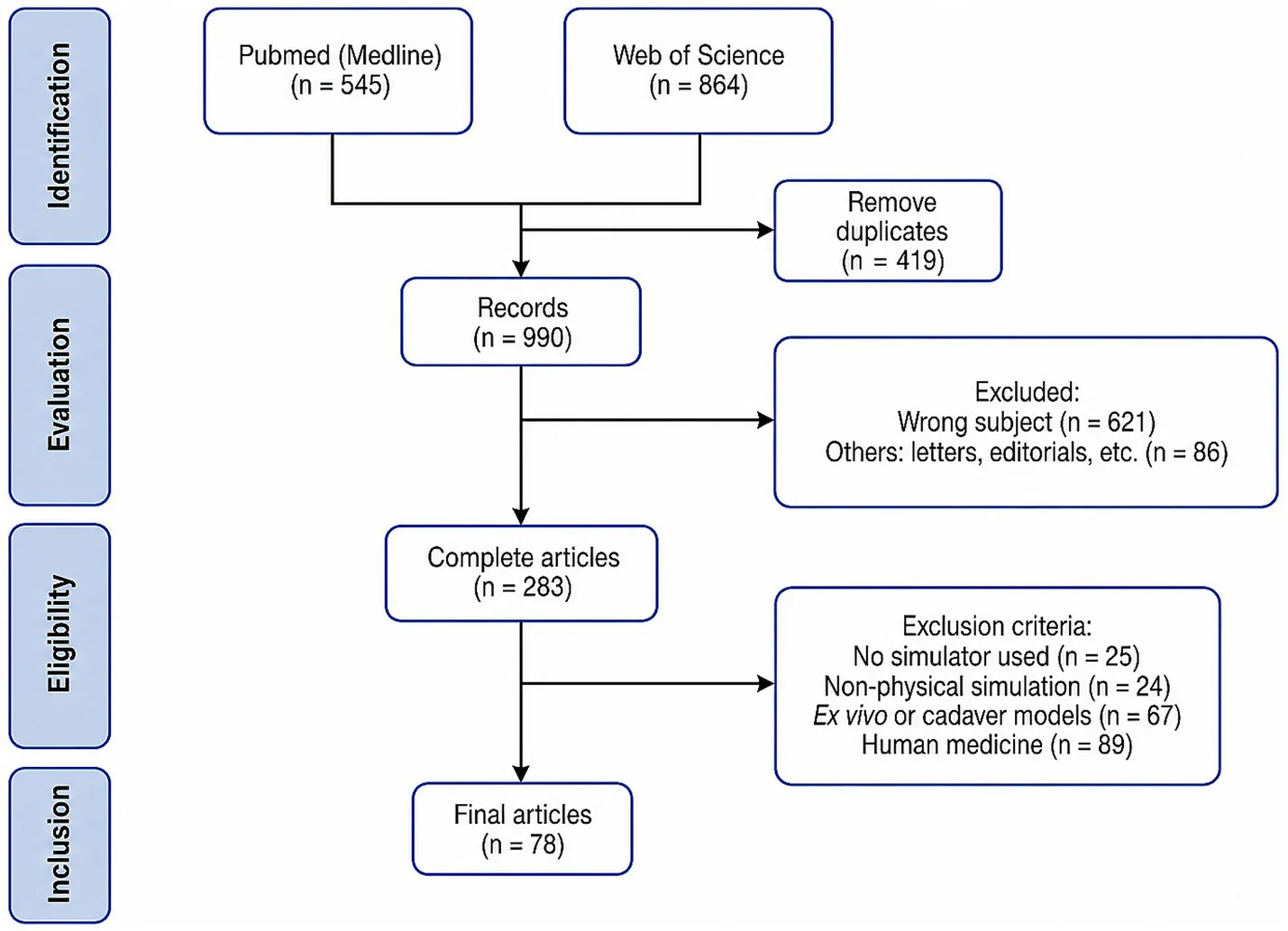
PRISMA flow chart.

A total of 545 and 864 publications were identified in PubMed and Web of Science databases, respectively. After removing duplicate entries, a total of 990 records were obtained and screened based on their titles and abstracts. Following the exclusion criteria, 707 records were evaluated and excluded as the subject did not comply with the goal of this review (*n* = 621) or the publication type was a review, letter, or similar (*n* = 86). The full text of the 283 articles remaining were analyzed, an additional 205 articles were excluded due to the absence of a simulator (*n* = 25), exclusive use of non-physical simulation (*n* = 24), reliance solely on ex vivo or cadaver models (*n* = 67), or focus on human medicine (*n* = 89). Ultimately, 78 studies were selected in the present study (Figure 1).

### 3.1 Simulator characteristic

**Figure 2:**
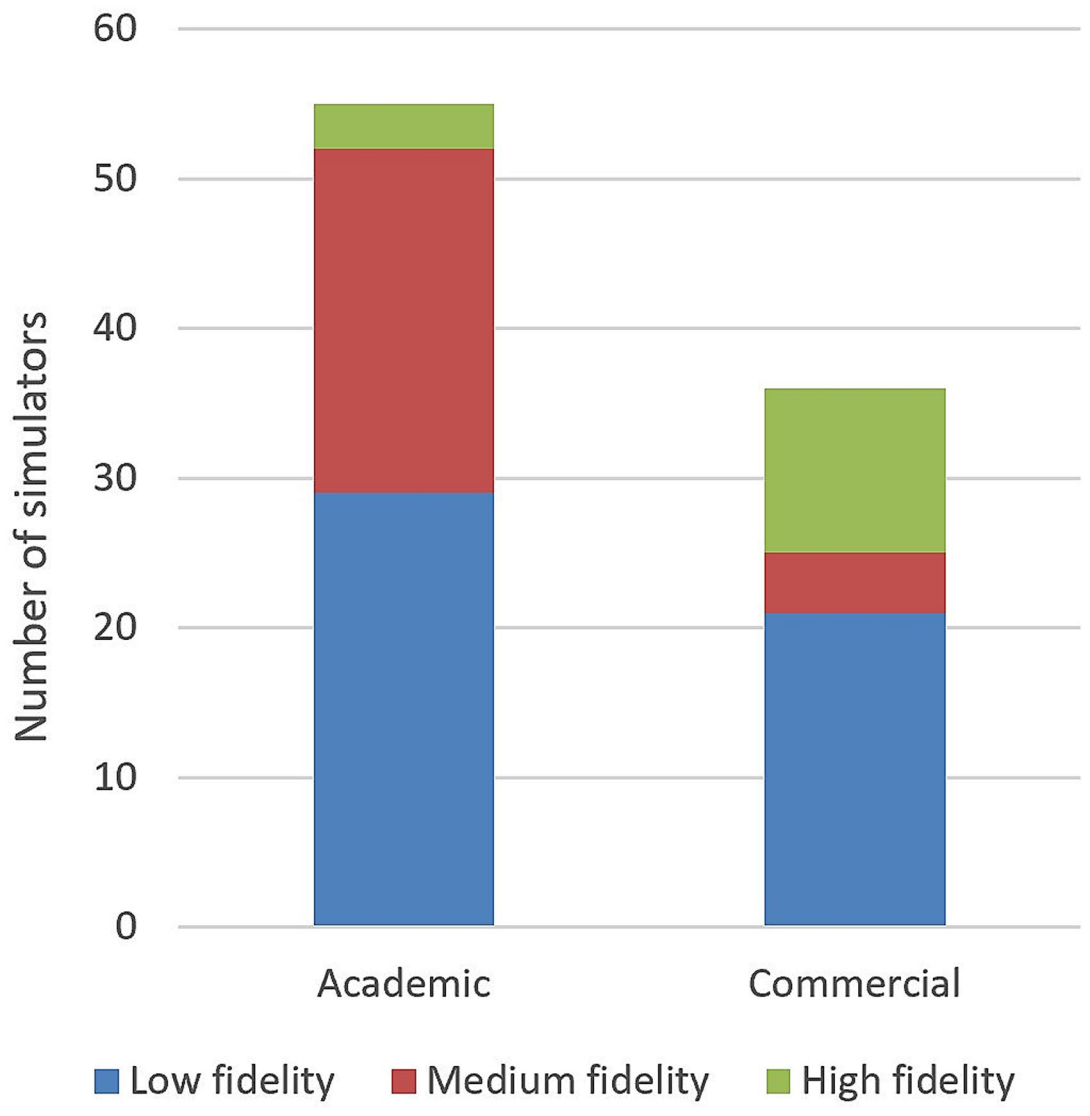
Distribution of simulator fidelity levels (low, medium, and high fidelity) by simulator type (academic and commercial).

Ninety-one different physical simulators for veterinary surgical training were analyzed in this study (Table 1). The included studies show a wide range of properties depending on the level of realism or the technique they aim to represent. A total of 50 simulators were classified as low fidelity (55%), 27 as medium fidelity (30%) and 14 as high fidelity (15%) (Figure 2). Regarding the development origin the findings were remarkably similar, with a higher predominance of academic models (*n* = 55) compared to commercial models (*n* = 36). In terms of task complexity, the number of advanced tasks (*n* = 54) used in the analyzed surgical simulator was higher than the basic ones (*n* = 37). Regarding the degree of realism and the origin of the identified simulators, most of the commercial simulators were classified as high fidelity (79% of the commercial simulators). In contrast, low- and medium-fidelity simulators predominantly originate from academic environments, accounting for 29 out of 50 (58%) and 23 out of 27 (85%), respectively.

**Table 1:** Results of the studies analyzed organized by educational and design characteristics of veterinary surgical simulators.

Article	Species	Anatomical region	Surgery approach	Tasks	Fidelity level	Scope of application	Simulator type	Participant	Evaluation method	Validation
Muehlberg et al. (2023) (51)	Bovine	Abdomen	Open surgery	Advanced	LF	STS	Academic	EXP, VS	Subjective (S), Subjective (IR)	FV, CV, CSV
Muehlberg et al. (2023) (51)	Bovine	Abdomen	Open surgery	Advanced	MF	STS	Academic	EXP, VS	Subjective (S), Subjective (IR)	FV, CV, CSV
Schank et al. (2025) (102)	Bovine	Abdomen	Open surgery	Advanced	MF	STS	Academic	VS, EXP	Subjective (IR), Subjective (S)	FV, CV, CSV
Anderson et al. (2021) (48)	Bovine	Genital	Open surgery	Advanced	MF	STS	Academic	EXP, VS	Objective, Subjective (S), Subjective (IR)	FV, CV, CSV, PV
Miller et al. (2026) (96)	Bovine	Genital	Open surgery	Advanced	MF	STS	Academic	EXP, VS	Subjective (S), Subjective (IR)	FV, CV, CSV
Brisson et al. (2023) (67)	Canine	Abdominal wall	Open surgery	Basic	LF	BSS	Commercial	VS	Subjective (IR)	
Brisson et al. (2025) (106)	Canine	Abdominal wall	Open surgery	Basic	LF	BSS	Commercial	VS	Objective, Subjective (S), Subjective (IR)	
Williamson et al. (2019) (119)	Canine	Abdominal wall	Open surgery	Basic	LF	BSS	Academic	EXP, VS	Subjective (S), Subjective (IR)	FV, CV, CSV
Williamson et al. (2019) (119)	Canine	Abdominal wall	Open surgery	Basic	HF	BSS	Academic	EXP, VS	Subjective (S), Subjective (IR)	FV, CV, CSV
Kim et al. (2025) (89)	Canine	Cardiovascular	Image-guided Surgery	Advanced	MF	IRAD	Academic	EXP	Objective, Subjective (S)	FV, CV
Lopez-Minguez et al. (2025) (95)	Canine	Cardiovascular	Image-guided Surgery	Advanced	LF	IRAD	Academic	VS, PIT	Objective, Subjective (S)	CV
Au Yong et al. (2019) (64)	Canine	Digestive	Open surgery	Advanced	HF	STS	Commercial	EXP, VS	Subjective (S)	FV, CV
Chen et al. (2017) (40)	Canine	Digestive	Laparoscopy	Advanced	HF	STS	Commercial	VS	Objective, Subjective (S), Subjective (IR)	CSV
Díaz-Regañón et al. (2024) (8)	Canine	Digestive	Endoscopy	Basic	MF	DIAG	Academic	VS	Subjective (S)	FV, CV, CCV
Grimes et al. (2019) (13)	Canine	Digestive	Open surgery	Advanced	HF	STS	Commercial	PIT, VS	Subjective (S)	FV, CV, CCV, PV
Turner et al. (2025) (117)	Canine	Digestive	Open surgery	Advanced	LF	STS	Academic	EXP, VS	Subjective (S)	FV, CV, CCV
Pe’re et al. (2025) (97)	Canine	Eye	Open surgery	Advanced	MF	STS	Academic	PIT, VS	Subjective (S)	FV, CV
Annandale et al. (2020) (61)	Canine	Genital	Open surgery	Advanced	HF	STS	Commercial	VS	Objective, Subjective (S), Subjective (IR)	FV, CV, CSV, PV
Chen et al. (2019) (37)	Canine	Genital	Laparoscopy	Advanced	HF	STS	Academic	EXP, PIT, VS	Objective, Subjective (S), Subjective (IR)	CSV, CV, FV
Erickson et al. (2023) (77)	Canine	Genital	Open surgery	Advanced	LF	STS	Academic	VS	Objective	
Erickson et al. (2026) (78)	Canine	Genital	Open surgery	Advanced	HF	STS	Commercial	VS	Objective	
Farrell et al. (2022) (79)	Canine	Genital	Open surgery	Advanced	LF	STS	Academic	VS, EXP	Subjective (IR), Subjective (S)	CV
García González et al. (2024) (1)	Canine	Genital	Open surgery	Advanced	MF	STS	Academic	VS	Objective	CSV
Hunt et al. (2020) (82)	Canine	Genital	Open surgery	Advanced	LF	STS	Academic	EXP, VS	Objective, Subjective (S), Subjective (IR)	FV, CV, CSV, PV
Hunt et al. (2023) (83)	Canine	Genital	Open surgery	Advanced	LF	STS	Academic	VS	Objective, Subjective (IR)	PV
Hunt et al. (2024) (84)	Canine	Genital	Open surgery	Advanced	LF	STS	Academic	VS	Subjective (IR)	CSV
Joonè et al. (2026) (85)	Canine	Genital	Open surgery	Advanced	LF	STS	Academic	VS	Objective	PV
Langebæk et al. (2020) (50)	Canine	Genital	Open surgery	Advanced	LF	STS	Academic	VS	Objective, Subjective (IR)	CSV
MacArthur et al. (2021) (5)	Canine	Genital	Open surgery	Advanced	LF	STS	Academic	VS	Subjective (S), Subjective (IR)	PV, CSV
Williamson et al. (2019) (118)	Canine	Genital	Open surgery	Advanced	LF	STS	Academic	EXP, VS	Subjective (S), Subjective (IR)	CSV
Zambelli et al. (2023) (121)	Canine	Genital	Open surgery	Advanced	LF	STS	Academic	VS, PIT	Subjective (S)	CV
Zambelli et al. (2023) (121)	Canine	Genital	Open surgery	Advanced	LF	STS	Academic	VS, PIT	Objective, Subjective (S)	CV
Langebæk et al. (2021) (92)	Canine	Head	Other	Advanced	MF	DIAG	Academic	EXP, VS	Subjective (S), Subjective (IR)	FV, CSV, CV
Sidhu et al. (2017) (44)	Canine	Head	Other	Advanced	MF	NS	Academic	EXP, PIT	Objective	CCV
Malek et al. (2020) (35)	Canine	Limb	Open surgery	Advanced	MF	OT	Academic	EXP	Objective, Subjective (S)	FV, CCV
Malek et al. (2020) (35)	Canine	Limb	Open surgery	Advanced	MF	OT	Commercial	EXP	Objective, Subjective (S)	FV, CCV
Carvalho et al. (2024) (70)	Canine	Mouth	Open surgery	Advanced	MF	BSS	Academic	EXP	Subjective (S)	FV, CV
Hunt et al. (2021) (26)	Canine	Mouth	Other	Basic	LF	BSS	Academic	EXP, VS	Objective, Subjective (IR), Subjective (S)	FV, CV, CCV
Hunt et al. (2021) (26)	Canine	Mouth	Other	Basic	MF	BSS	Academic	EXP, VS	Objective, Subjective (IR), Subjective (S)	FV, CV, CCV
Hunt et al. (2021) (26)	Canine	Mouth	Other	Basic	HF	BSS	Commercial	EXP, VS	Objective, Subjective (IR), Subjective (S)	FV, CV, CCV
Laws et al. (2024) (93)	Canine	Skin	Open surgery	Advanced	HF	STS	Academic	EXP, PIT, VS	Subjective (S)	FV, CV
Cruz et al. (2024) (71)	Canine	Urinary	Open surgery	Advanced	MF	STS	Academic	VS	Subjective (S)	FV, CV
Saunders et al. (2017) (101)	Canine	Vascular	Other	Advanced	MF	STS	Academic	EXP, PIT	Subjective (S)	FV, CV
Braid (2025) (66)	Equine	Genital	Open surgery	Advanced	LF	STS	Academic	EXP, VS	Subjective (S)	FV, CV
Devine et al. (2024) (75)	Equine	Genital	Open surgery	Advanced	LF	STS	Academic	VS, EXP	Subjective (IR), Subjective (S)	CV, CSV
Elarbi et al. (2018) (76)	Equine	Genital	Laparoscopy	Advanced	MF	STS	Academic	EXP, VS	Objective, Subjective (S)	CCV, CSV, CV, FV
Sheats et al. (2021) (52)	Equine	Genital	Open surgery	Advanced	LF	STS	Academic	EXP, VS	Subjective (S), Subjective (IR)	CV, CSV, PV
Beaulieu et al. (2022) (59)	Equine	Limb	Other	Advanced	MF	DIAG	Academic	EXP, PIT	Objective, Subjective (S)	FV, CSV, CV
Aly et al. (2023) (63)	Feline	Genital	Open surgery	Advanced	HF	STS	Commercial	VS	Objective, Subjective (S)	CSV, PV
Zambelli et al. (2023) (121)	Feline	Genital	Open surgery	Advanced	LF	STS	Academic	VS, PIT	Subjective (S)	CV
Phillips et al. (2018) (100)	Feline	Urinary	Open surgery	Advanced	MF	STS	Academic	EXP, PIT	Subjective (S)	FV, CV
Azuma and Monnet (2026) (104)	Human	Digestive	Laparoscopy	Advanced	HF	STS	Commercial	PIT	Objective	
Danner et al. (2026) (72)	Human (with veterinary applications)	Digestive	Laparoscopy	Advanced	HF	STS	Commercial	EXP, PIT	Subjective (S), Subjective (IR)	FV, CV, CSV
Forman et al. (2026) (108)	Human (with veterinary applications)	Genital	Laparoscopy	Advanced	HF	STS	Commercial	VS	Objective, Subjective (IR)	PV
French et al. (2021) (62)	Human (with veterinary applications)	Genital	Laparoscopy	Advanced	HF	STS	Commercial	VS	Objective, Subjective (S), Subjective (IR)	FV, CV, PV
Dejescu et al. (2025) (74)	Rabbit	Genital	Laparoscopy	Advanced	MF	STS	Academic	VS	Subjective (S)	FV, CV
Bainier et al. (2021) (65)	Rodent	Head	Other	Advanced	MF	NS	Academic	EXP	Subjective (S)	FV, CV
Knoll et al. (2025) (111)	Tortoise	Other	Open surgery	Advanced	MF	STS	Academic	VS, EXP	Subjective (IR), Subjective (S)	FV, CV
Klonner et al. (2019) (90)	Undefined	Abdominal wall	Open surgery	Basic	LF	BSS	Academic	EXP, PIT	Objective	CSV
Caston et al. (2016) (107)	Undefined	Digestive	Open surgery	Advanced	MF	BSS	Commercial	VS	Objective, Subjective (IR)	CSV
Grimes et al. (2019) (13)	Undefined	Digestive	Open surgery	Advanced	MF	STS	Commercial	PIT, VS	Subjective (S)	FV, CV, CCV, PV
Perez-Rivero et al. (2022) (98)	Undefined	Genital	Open surgery	Advanced	LF	STS	Academic	VS	Objective	
McGowan et al. (2023) (113)	Undefined	Skin	Other	Basic	MF	STS	Academic	VS	Objective, Subjective (S), Subjective (IR)	CV, CSV
Baillie et al. (2020) (45)	Undefined	Undefined	Open surgery	Basic	LF	BSS	Academic	EXP, VS	Objective, Subjective (S), Subjective (IR)	FV, CV, CSV, CCV
Baillie et al. (2020) (45)	Undefined	Undefined	Open surgery	Basic	LF	BSS	Academic	EXP, VS	Objective, Subjective (S), Subjective (IR)	FV, CV, CSV, CCV
Bakıcı et al. (2026) (105)	Undefined	Undefined	Other	Basic	LF	BSS	Commercial	VS	Subjective (IR)	
Decloedt et al. (2021) (73)	Undefined	Undefined	Open surgery	Basic	LF	BSS	Commercial	VS	Subjective (IR)	
Giusto et al. (2018) (81)	Undefined	Undefined	Other	Basic	LF	BSS	Academic	VS	Objective, Subjective (S)	
Kuzminsky et al. (2023) (91)	Undefined	Undefined	Other	Basic	MF	BSS	Academic	VS	Objective, Subjective (IR)	
Meritet et al. (2021) (114)	Undefined	Undefined	Other	Basic	LF	BSS	Academic	VS	Objective, Subjective (S)	CSV
Nocera et al. (2024) (116)	Undefined	Undefined	Open surgery	Basic	LF	BSS	Commercial	VS	Subjective (S), Subjective (IR)	CV
Pérez-Rivero et al. (2026) (99)	Undefined	Undefined	Open surgery	Basic	LF	BSS	Academic	VS	Objective	CV
Austin and Fransson (2025) (103)	Undefined (box trainer)	Undefined (box trainer)	Laparoscopy	Basic	LF	BSS	Commercial	EXP, PIT, VS	Objective	CSV
Buote et al. (2023) (68)	Undefined (Box Trainer)	Undefined (box trainer)	Robotic Surgery	Basic	LF	BSS	Commercial	VS	Objective	
Buote et al. (2024) (69)	Undefined (box trainer)	Undefined (box trainer)	Robotic Surgery	Basic	LF	BSS	Commercial	EXP, PIT	Objective	
Chen et al. (2017) (40)	Undefined (box trainer)	Undefined (box trainer)	Laparoscopy	Basic	MF	STS	Commercial	VS	Objective, Subjective (S), Subjective (IR)	CSV
Chen et al. (2019) (37)	Undefined (box trainer)	Undefined (box trainer)	Laparoscopy	Basic	LF	STS	Commercial	EXP, PIT, VS	Objective, Subjective (S), Subjective (IR)	CSV, CV, FV
Elarbi et al. (2018) (76)	Undefined (box trainer)	Undefined (box trainer)	Laparoscopy	Basic	LF	BSS	Commercial	EXP, VS	Objective, Subjective (S)	CCV, CSV, CV, FV
Forman et al. (2026) (108)	Undefined (box trainer)	Undefined (box trainer)	Laparoscopy	Basic	LF	BSS	Commercial	VS	Objective, Subjective (IR)	PV
Fransson et al. (2023) (80)	Undefined (box trainer)	Undefined (box trainer)	Laparoscopy	Advanced	LF	STS	Commercial	EXP, PIT	Objective	CSV, PV
Hincapié-Gutiérrez et al. (2023) (42)	Undefined (box trainer)	Undefined (box trainer)	Laparoscopy	Basic	LF	BSS	Academic	EXP, PIT	Objective, Subjective (S), Subjective (IR)	FV, CV, CSV
Hottmann and Fransson (2024) (109)	Undefined (box trainer)	Undefined (box trainer)	Laparoscopy	Basic	LF	BSS	Commercial	VS	Objective	CCV
Kilkenny et al. (2017) (110)	Undefined (box trainer)	Undefined (box trainer)	Laparoscopy	Basic	LF	BSS	Commercial	VS	Objective, Subjective (S)	
Kilkenny et al. (2017) (86)	Undefined (box trainer)	Undefined (box trainer)	Laparoscopy	Basic	LF	BSS	Commercial	VS	Objective, Subjective (IR)	
Kilkenny et al. (2017) (87)	Undefined (box trainer)	Undefined (box trainer)	Laparoscopy	Basic	LF	BSS	Commercial	VS, VTECH	Objective, Subjective (S)	CSV
Kilkenny et al. (2019) (88)	Undefined (box trainer)	Undefined (box trainer)	Laparoscopy	Basic	LF	BSS	Commercial	VS	Objective	
Lencioni et al. (2017) (94)	Undefined (box trainer)	Undefined (box trainer)	Laparoscopy	Basic	LF	BSS	Commercial	VS	Objective	
Levi et al. (2019) (112)	Undefined (box trainer)	Undefined (box trainer)	Laparoscopy	Basic	LF	BSS	Commercial	VS	Objective	CSV
Micsa et al. (2023) (115)	Undefined (box trainer)	Undefined (box trainer)	Laparoscopy	Basic	LF	BSS	Academic	VS	Objective	
Strohmeier et al. (2021) (43)	Undefined (box trainer)	Undefined (box trainer)	Laparoscopy	Basic	LF	BSS	Commercial	PIT, VS	Objective, Subjective (S)	CV, CCV
Yun and Lee (2026) (120)	Undefined (box trainer)	Undefined (box trainer)	Laparoscopy	Basic	LF	BSS	Commercial	VS	Objective, Subjective (S)	

Fidelity level: HF, high fidelity; LF, low fidelity; MF, medium fidelity.

Scope of application: BSS, basic surgical skills; DIAG, diagnostic procedures; IRAD, interventional radiology; NS, neurosurgery; OT, orthopedics and traumatology; STS, soft tissue surgery.

Participant: EXP, experienced professionals; PIT, professionals in training; VS, veterinary students; VTECH, veterinary technicians.

Evaluation method: IR, instructor rating; S, survey.

Validation: CCV, concurrent validity; CSV, construct validity; CV, content validity; FV, face validity; PV, predictive validity.

### 3.2 Surgical scope

**Figure 3:**
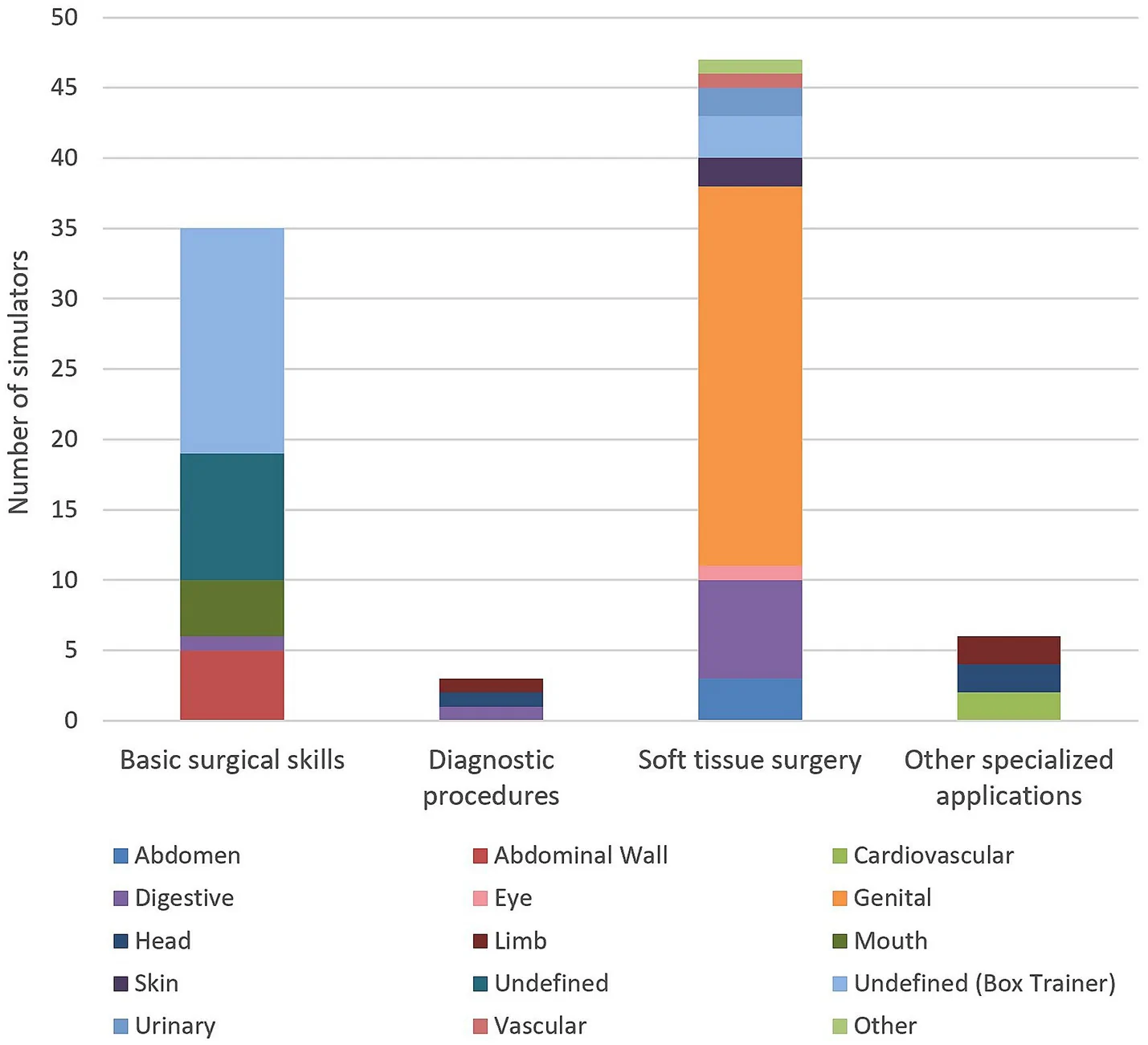
Distribution of anatomical region by scope of application.

Considering the animal species that the simulator aims to represent, most results focused on the canine model (42%), while 21% were box trainers (undefined). In terms of surgical approach, the data highlighted two main categories: open surgery (*n* = 48) and laparoscopy (*n* = 25). Considering the anatomical region (Figure 3), the distribution revealed a wide range of systems but was mainly represented by genital models (30%), digestive models (10%), and abdominal wall (5%), with comparable results for the remaining categories. Ten percent of simulators did not indicate a specific anatomical region (undefined). Regarding the scope of application, the proportions of results were similar between simulators for soft tissue surgery (*n* = 47) and simulators for basic surgical skills training (*n* = 35).

### 3.3 Evaluation framework

**Figure 4:**
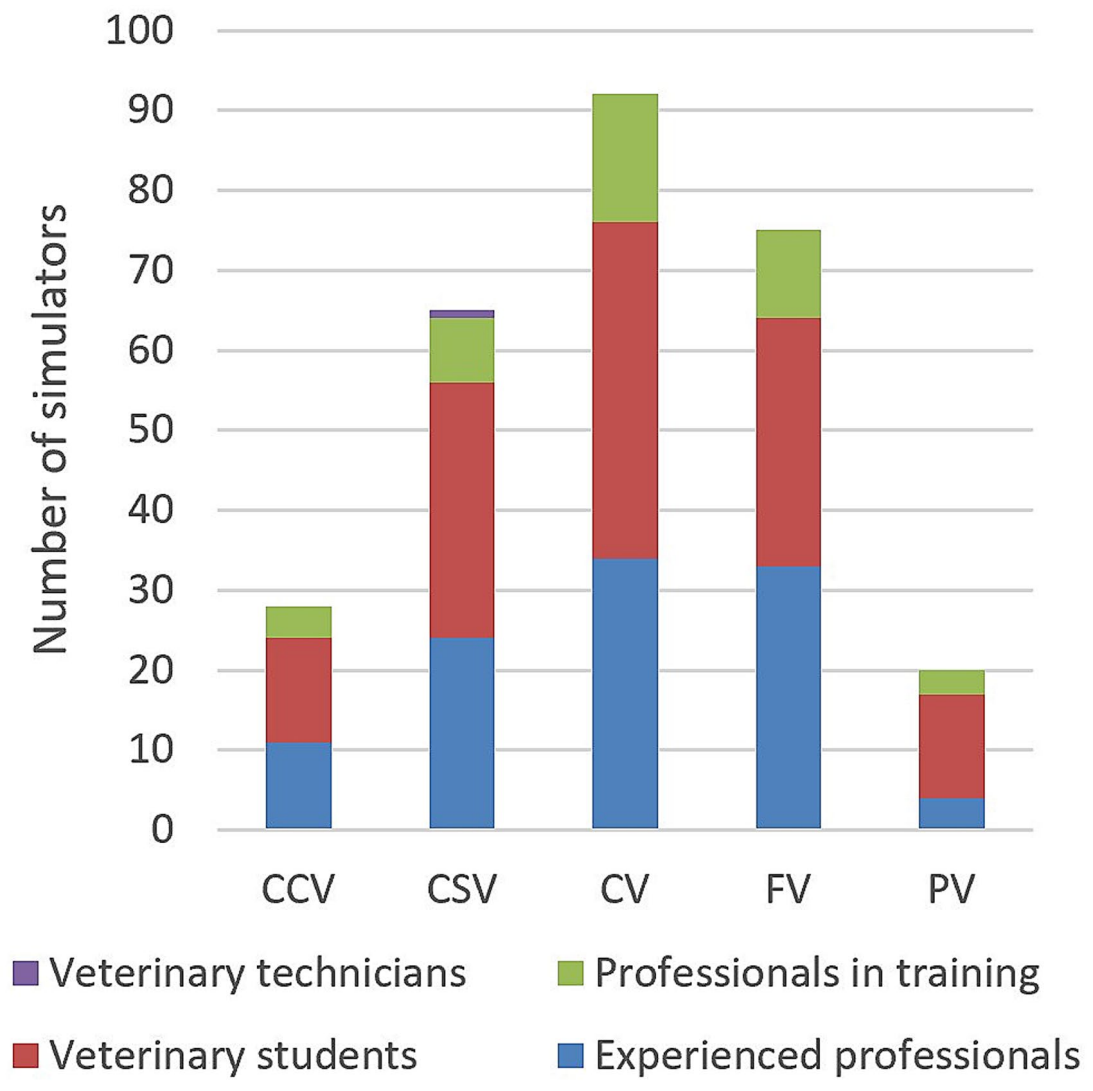
Distribution of participants by validation (CCV, concurrent validity; CSV, construct validity; CV, content validity; FV, face validity; PV, predictive validity).

The evaluation of simulators was conducted across the studies using a variety of participants, methodologies, and validations strategies with heterogeneous approaches. The primary users in these validation studies were veterinary students (*n* = 131), with additional involvement of experienced professionals (*n* = 106), and professionals in training (*n* = 42) (Figure 4). The findings on evaluation methods were consistent, with surveys being the most usual form of subjective assessment (*n* = 62), followed by objective measures (*n* = 56), and, to a lesser extent, instructor ratings as subjective assessment (*n* = 42). Different validation methods were applied, with content validity (*n* = 92) and face validity (*n* = 75) being the most frequently reported. These were accompanied by construct validity (*n* = 65), while concurrent validity (*n* = 28) and predictive validity (*n* = 20) were the least frequently performed validations.

### 3.4 Risk of bias assessment

**Figure 5:**
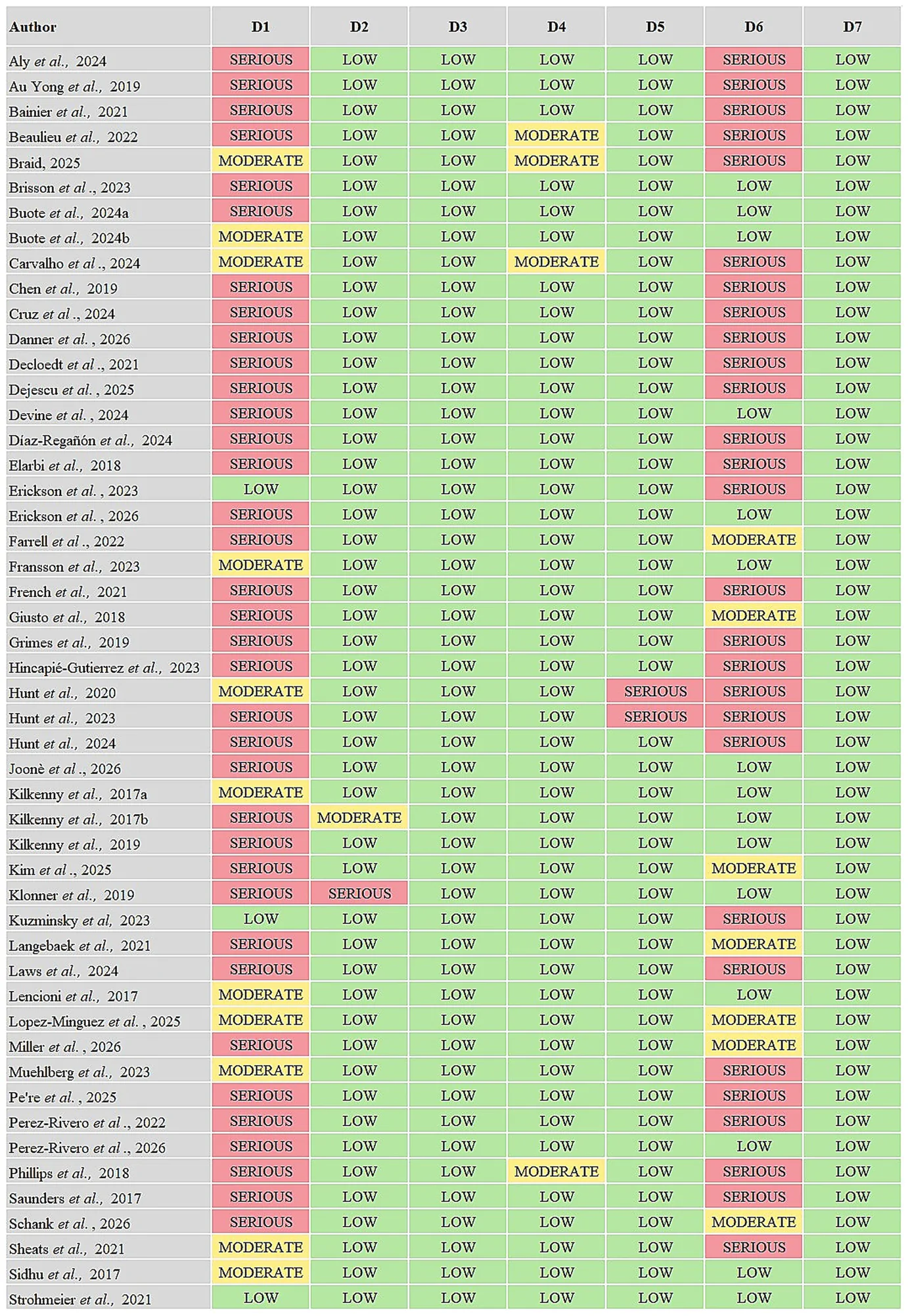
Bias risk analysis using the ROBINS-I methodology with regard to seven domains: D1 (bias due to confounding); D2 (bias in selection of participants into the study); D3 (bias in classification of interventions); D4 (bias due to deviations from intended interventions); D5 (bias due to missing data); D6 (bias in measurement of outcomes); and D7 (bias in selection of the reported result). Each item is rated as ‘LOW’ (low concern for bias), ‘MODERATE’ (non-negligible bias with limited impact on causal interpretation), ‘SERIOUS’ (substantial bias likely to meaningfully affect the estimated effect), and ‘CRITICAL’ (bias of sufficient magnitude to render the study unsuitable for credible effect estimation). Studies analyzed: Aly et al. 2023 (63); Au Yong et al. 2019 (64); Bainier et al. 2021 (65); Beaulieu et al. 2022 (59); Braid, 2025 (66); Brisson et al. 2023 (67); Buote et al. 2024 (68); Buote et al. 2024 (69); Carvalho et al. 2024 (70); Chen et al. 2019 (37); Cruz et al. 2024 (71); Danner et al. 2026 (72); Decloedt et al. 2021 (73); Dejescu et al. 2025 (74); Devine et al. 2024 (75); Díaz-Regañón et al. 2024 (8); Elarbi et al. 2018 (76); Erickson et al. 2023 (77); Erickson et al. 2026 (78); Farrell et al. 2022 (79); Fransson et al. 2023 (80); French et al. 2021 (62); Giusto et al. 2018 (81); Grimes et al. 2019 (13); Hincapié-Gutiérrez et al. 2023 (42); Hunt et al. 2020 (82); Hunt et al. 2023 (83); Hunt et al. 2024 (84); Joonè et al. 2026 (85); Kilkenny et al. 2017 (86); Kilkenny et al. 2017 (87); Kilkenny et al. 2019 (88); Kim et al. 2025 (89); Klonner et al. 2019 (90); Kuzminsky et al. 2023 (91); Langebaek et al. 2021 (92); Laws et al. 2024 (93); Lencioni et al. 2017 (94); Lopez-Minguez et al. 2025 (95); Miller et al. 2026 (96); Muehlberg et al. 2023 (51); Pe’re et al. 2025 (97); Perez-Rivero et al. 2022 (98); Perez-Rivero et al. 2026 (99); Phillips et al. 2018 (100); Saunders et al. 2017 (101); Schank et al. 2026 (102); Sheats et al. 2021 (52); Sidhu et al. 2017 (44); Strohmeier et al. 2021 (43).

Concerning the assessment of risk of bias, ROBINS-I indicated an overall low risk of bias in most of the domains analyzed (Figure 5). Nevertheless, two domains were identified as particularly compromised: bias due to confounding (domain 1) and bias in measurement of outcomes (domain 6). For bias due to confounding factors, a predominantly serious risk of bias was assigned, mainly due to insufficient control of relevant variables such as participants’ prior experience, skill level, or learning curve effects. To a lesser extent, nine articles were considered moderate, indicating that although confounding factors existed, they were adequately measured. For bias in measurement of outcomes, a serious risk of bias was also observed in most studies, primarily because subjective outcomes were employed, thereby increasing the likelihood of measurement bias. The remaining domains showed greater methodological consistency and were predominantly classified as low risk.

**Figure 6:**
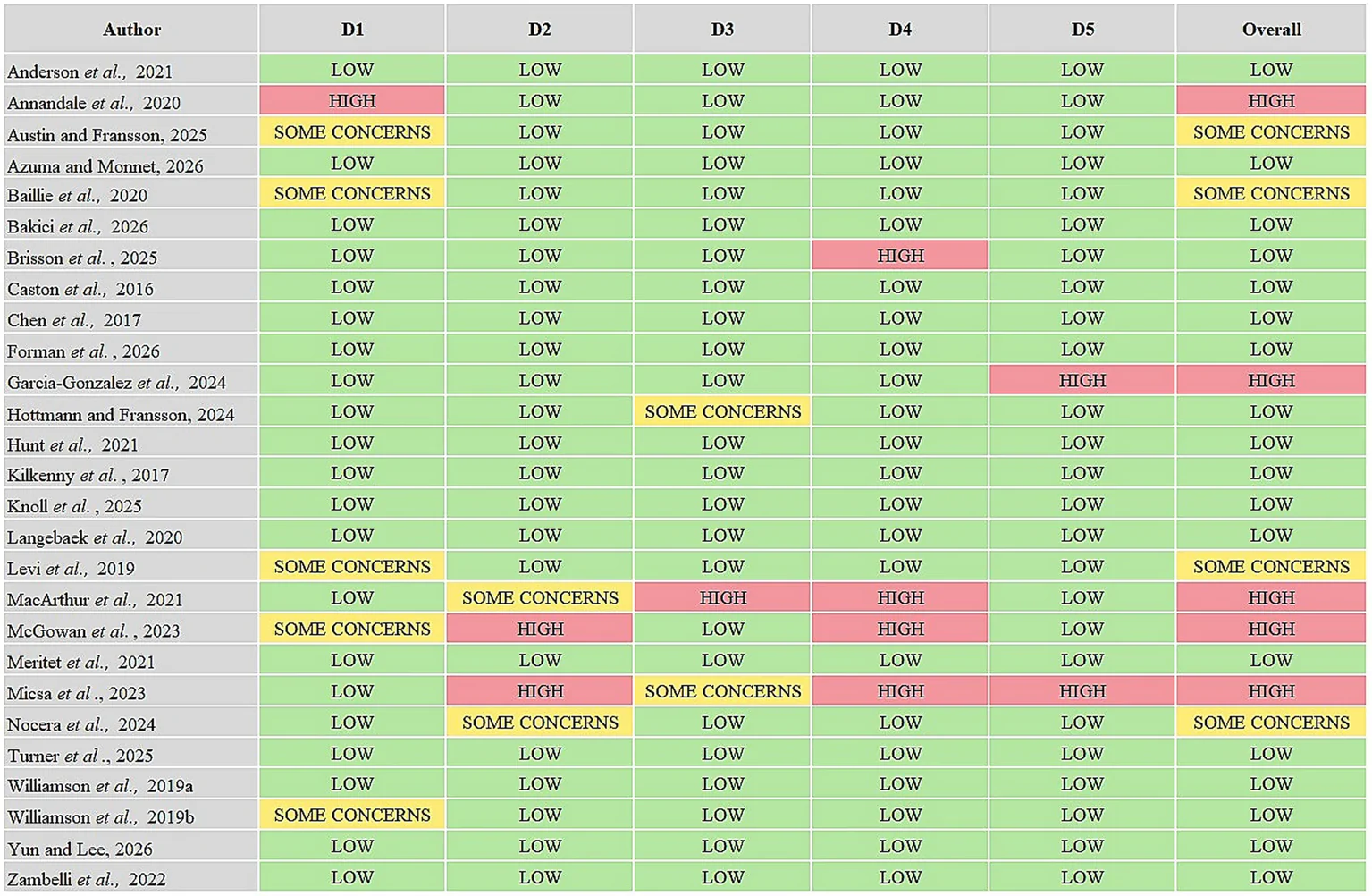
Bias risk analysis using the RoB 2.0 methodology considering five domains: D1 (bias arising from the randomization process); D2 (bias due to deviations from intended interventions); D3 (bias due to missing outcome data); D4 (bias in measurement of the outcome); and D5 (bias in selection of the reported result). Each item is rated as ‘LOW’ (low risk of bias), ‘HIGH’ (high risk of bias), or ‘SOME CONCERNS’ (methodological limitations that introduce uncertainty in the risk of bias). Studies analyzed: Anderson et al. 2021 (48); Annandale et al. 2020 (61); Austin and Fransson, 2025 (103); Azuma and Monnet, 2026 (104); Baillie et al. 2020 (45); Bakici et al. 2026 (105); Brisson et al. 2025 (106); Caston et al. 2016 (107); Chen et al. 2017 (40); Forman et al. 2026 (108); Garcia-Gonzalez et al. 2024 (1); Hottmann and Fransson, 2024 (109); Hunt et al. 2021 (26); Kilkenny et al. 2017 (110); Knoll et al. 2025 (111); Langebaek et al. 2020 (50); Levi et al. 2019 (112); MacArthur et al. 2021 (5); McGowan et al. 2023 (113); Meritet et al. 2021 (114); Micsa et al. 2023 (115); Nocera et al. 2024 (116); Turner et al. 2025 (117); Williamson et al. 2019 (118); Williamson et al. 2019 (119); Yun and Lee, 2026 (120); Zambelli et al. 2022 (121).

In the assessment of risk of bias in randomized studies, the use of RoB 2.0 tool showed that most studies had an overall low risk of bias, with only a limited proportion classified as having some concerns or high risk of bias (Figure 6). The main signs of risk of bias were concentrated in domains 1 and 4, where some studies were rated as having some concerns or high risk due to issues related to the randomization sequence, lack of adequate blinding, or the measurement of the outcome. One article was not assessed for risk of bias due to its descriptive nature and absence of surgical interventions [Malek et al. (35)].

## 4 Discussion

The availability of reliable, useful and properly validated training tools may be essential to ensure adequate skill acquisition during the initial stages of veterinary surgery without the need to resort to cadaveric or *in vivo* models. This systematic review has conducted a comprehensive analysis of the various simulation options for training in veterinary surgery, both commercial and prototype versions, which has provided an overview of their current levels of fidelity, the validations they have undergone and the current limitations that would need to be addressed in future work.

The main findings of the present systematic review suggest a predominance of simulators for veterinary surgical training that generally exhibit low fidelity and appear to be focused on a limited range of anatomical regions and species. On the one hand, it appears to be a greater representation of canine models simulating the genital tract, primarily intended for sterilization procedures, as this is one of the most common surgical interventions in veterinary practice (36). In addition, a large number of studies reported the use of box trainers, intended for general learning of basic laparoscopic surgical skills, a process closely related to the learning curve of minimally invasive surgery and requiring the acquisition of specific technical skills (37). In this regard, the comparison between surgical approach and task complexity suggested that basic tasks were predominantly focused on laparoscopic training which may reflect the need to initiate technical skill acquisition in box trainers to address the steep learning curve associated with minimally invasive surgery (38). In contrast, advanced tasks appeared to be more frequently focused on open surgery training, concentrating on the training of procedure-specific surgical techniques.

The high cost associated with the development and production of highly realistic models, together with their technical complexity, may explain why this type of simulator is mainly commercial. On the other hand, low-fidelity models are usually easier and more affordable to manufacture. As for commercial simulators, a review by Hunt et al., the prices of 36 simulators were analyzed, ranging from US$10 for basic suturing simulators to over US$30,000 for the most advanced systems, which incorporate haptic feedback, physiological responses, and multiple integrated surgical procedures (26). Moreover, one of the included studies used a human casing simulator. As per inclusion criteria of the present review, it was deemed eligible for analysis as the internal artificial structures were synthetic organs based on canine anatomy. The surgical procedure performed was ovariectomy and the participants were veterinary students, which may support its relevance within a veterinary surgical training context. These results illustrate the wide variability in the simulator market and may suggest a direct relationship between the level of realism, versatility, and functionality and the financial investment required for their development and, consequently, their commercialization cost.

Regarding the validation of surgical training simulators, it should be noted that no clear unified or standardized strategy has been identified in the included studies. Therefore, in this systematic review, the types of validation were classified according to the frameworks proposed in the scientific literature (12, 27–29). As many of the models evaluated did not undergo a formal validation process, it was considered necessary to establish equivalence based on the characteristics described by the aforementioned authors.

With regard to face and content validity, numerous studies appear to have relied on evaluations performed by students. While such assessments may provide preliminary insights, robust validation may require the involvement of experts with appropriate technical and anatomical expertise. Gallagher et al. describe both as subjective assessment, but emphasize that content validity should be evaluated by experts. The results of our study show that 58 of the 92 records assessing content validity (63%) did not include veterinary experts as evaluators, and may introduce bias and compromise the validity of the findings because the evaluators’ limited experience (29). In contrast, other studies incorporated expert assessors who delivered structured feedback regarding the simulator’s realism and its capacity to train the intended skills suggesting that such feedback is essential when validating a simulator as an educational tool. For example, Nursanti et al. used an expert panel scored by a five-point Likert questionnaire, specifically developed for ocular surgery (39).

Construct validity can be addressed through different methodological designs. One approach involves dividing novice participants into two groups, providing structured training to one and subsequently comparing performance differences attributable to the training simulator (40). A more methodologically rigorous strategy is to compare participants with clearly differentiated levels of expertise, typically contrasting inexperienced individuals with experienced surgeons (41). This approach may enable a more robust evaluation of the simulator’s ability to discriminate between distinct levels of surgical competence through validated assessment methods such as the Objective Structured Assessment of Technical Skills (OSATS) (42) or McGill Inanimate System for Training and Evaluation of Laparoscopic Skills (MISTELS) (43).

Although concurrent validity may be established by developing and comparing two handcrafted simulators (44, 45) or by comparing performance across two commercial simulators (13, 46), a potentially more robust approach may consist of comparing a homemade simulator with a commercial counterpart (26, 43, 47). In this scenario the commercial simulator is frequently used as an external reference. However, this does not inherently imply technical superiority. The analytical focus should therefore perhaps be directed toward the standardization and comparability of performance metrics. For this reason, a rigorous validation process may be essential to establish surgical simulators as reliable training tools. In some cases, predictive validation of the developed simulator was also included, involving students with comparable levels of training. This design allowed for the assessment of skill acquisition and demonstrated potential improvements in performance during actual clinical procedures following prior simulator-based training (13, 47, 48).

The assessment of risk of bias provides an important context for interpreting the results of this review. As expected, non-randomized studies showed greater methodological heterogeneity with recurring limitations related to confounding and outcome measurement. In particular, insufficient control of initial classification parameters, such as prior experience, skill level, or learning effects, and the frequent use of subjective outcomes were the main sources of bias. Conversely, the randomized studies evaluated demonstrated good methodological quality, with a predominantly low risk of bias in most areas, supporting the internal validity of their results. The main limitations centered on the transparency of the randomization process and the selection of reported outcomes. While these limitations do not invalidate the observed trends, they reduce confidence in causal inference and highlight the need for cautious interpretation. Taken together, these findings emphasize the importance of rigorous study design, objective outcome measures, adequate randomization of study groups, and transparency in reporting to strengthen the evidence base for future research on simulation-based surgical training.

Regarding the educational and clinical implications of the findings, simulation could play a fundamental role in undergraduate training. Simulators may be essential for students to practice and learn basic surgical skills and techniques. In this context, simulation complements theoretical teaching and aligns with the principles of the 3 Rs, as it allows for repeated practice and refinement of skills, enabling students to enter clinical rotations and practical training better prepared than with traditional educational methods (2).

In contrast, postgraduate training and surgical residency programs appear to reveal a clear shortage of high-fidelity simulators specifically designed for advanced and specialized procedures, such as advanced laparoscopy, thoracic surgery, or neurosurgery. This may be attributed to the smaller target market and the greater complexity and higher development costs involved. The availability of high-fidelity models in these areas could substantially improve learning curves, reduce technical errors, and facilitate a safer and more effective transition to actual clinical practice (4). However, patient safety could be significantly improved by implementing standardized simulators that define minimum competency thresholds before performing procedures on live animals. This approach can also contribute to greater consistency in surgical training across academic institutions and veterinary hospitals, promoting more consistent educational standards and clinical outcomes (17, 18, 49).

This systematic review has some limitations that should be considered when interpreting its findings. Although the 10-year search period was selected to focus on recent developments, earlier relevant studies may have been omitted. Similarly, searching on only two bibliographic databases may have excluded studies indexed elsewhere.

Furthermore, the methodological appraisal of the included studies should not be interpreted as a comprehensive assessment of overall study quality. The focus of RoB 2.0 and ROBINS-I was on evaluation of potential threats to internal validity, taking into account that some studies presenting low risk of bias may still have had methodological or reporting limitations that affected the reliability, comparability and generalizability of the findings. Despite these limitations, the present review provides a useful and updated insight into the characteristics of veterinary surgical simulators.

Previous reviews by Hunt et al. and Braid (14, 15) provided valuable analyses and classifications of simulators used in veterinary education, while the present study offers an updated synthesis to identify patterns, limitations, and research gaps, in accordance with the PRISMA statement. Furthermore, although two recent systematic reviews by Veenema et al. and Noyes et al. (17, 18) have addressed simulation in veterinary education, their analyses were predominantly pedagogical and quantitative. Unlike these studies, the present review applies a systematic framework for data extraction and classification in order to specifically examine the technical characteristics, validation processes, and surgical applications of simulators, providing a comprehensive and reproducible overview of the current state of veterinary surgical simulation.

One of the main limitations of the studies analyzed was the predominance of canine models, with limited representation of other species, that may limit the extrapolation and applicability of simulators to veterinary practice as a whole. This lack of diversity could reduce the educational potential of the models and highlights the need for simulators adapted to other species of clinical and surgical interest. Additionally, a considerable proportion of the simulators identified had a low degree of realism, as they were mostly made from common and reused materials, such as Foley catheters. Limited financial resources in academic settings, coupled with the high cost of developing higher-fidelity models or purchasing commercially available simulators, significantly restrict the development and implementation of higher-fidelity models, favoring the use of basic low-cost simulators.

Several studies have shown that low-fidelity simulators, when integrated as a complement to traditional teaching methods, seem to provide significant educational benefits. For instance, Langebæk et al. reported that the manufacture of simulators by surgery students using accessible materials was associated with increased confidence, active participation, and opportunities for anatomical training and repetitive practice in educational settings (50). Similarly, other studies have shown the development of low- to medium-fidelity simulators using inexpensive materials, applicable to a wide range of surgical techniques and species. This may suggest that functional and reproducible training tools for veterinary surgical practice can be generated without necessarily relying on high-cost commercial devices (1, 51, 52).

Despite their absence in the results of the present review, porcine models are of considerable interest in the context of surgical research and training, particularly in human medicine, due to their anatomical and physiological similarity to humans. These models are widely employed across multiple surgical fields and specialties (53–56). To a lesser extent, they are also used in veterinary education where ethical considerations have increasingly driven the replacement of canine models with porcine alternatives, enabling surgical training in a highly realistic setting (13, 57, 58). The development of artificial models based on porcine anatomy appears to represent a highly impactful line of research aimed at reducing the learning curve of veterinary surgeons. Furthermore, the implementation of these innovative solutions may help minimize the risk of errors and handling-related accidents during experimental practice, while promoting animal welfare in accordance with the principles of Replacement, Reduction, and Refinement (the 3Rs) (22).

Regarding the surgical specialties addressed, orthopedics and traumatology remained relatively underrepresented despite their clinical relevance in veterinary practice. This is a significant area of surgical pathologies in routine clinical practice, including cranial cruciate ligament rupture, patellar luxation, and hip dislocation (59, 60). This may be due to the high morphological variability between and within species, which could make it difficult to develop universal simulators with cross-sectional validity.

Another critical issue highlighted by this review is the absence of methodologically robust validation of the simulators analyzed. This lack of methodological rigor necessitated the assignment of validation types based on certain characteristics described by the authors, even when these did not conform to standardized validation frameworks. In numerous studies, assessments of simulator realism or educational utility were conducted by students or participants with limited prior experience, thereby introducing a substantial risk of measurement bias (8, 61, 62). This practice raises concerns regarding the external validity of the findings and restricts the ability to draw firm conclusions about the true effectiveness of the models. Collectively, these limitations underscore the need for more rigorous validation strategies, incorporating evaluators with varying levels of expertise and objective performance metrics in order to strengthen the scientific evidence base in the field of veterinary simulation. Furthermore, the validation processes were heterogeneous and inconsistent, as only a subset of the included articles provided methodologically sound evidence. The marked variability in study design, methodology, and reported results made it difficult to classify the studies into homogeneous categories, which gave this systematic review a predominantly descriptive character and justified the absence of advanced statistical analyses.

Building upon the identified limitations and the trends observed in the current body of evidence, several directions for future research can be proposed to advance the field of veterinary surgical simulation. A remarkable proportion of studies have focused on simulated procedures corresponding to low-complexity techniques, such as suturing or sterilization. Although the latter was classified as an advanced technique in this review due to its surgical nature, it nevertheless represents a routine and fundamentally basic procedure within daily clinical practice.

In this context, there is a clear need to develop simulators capable of reproducing more complex surgical procedures that require specialized training, such as hepatic resections, urological interventions, and orthopedic surgery. These procedures pose significant clinical challenges, yet structured simulation-based training in these domains remains limited. The development of such solutions would provide substantial added value and constitute an important educational tool to support the acquisition of advanced surgical skills in veterinary medicine.

## 5 Conclusion

This systematic review provides a comprehensive overview of the current landscape of physical simulators used in veterinary surgical education. The reviewed evidence supports the educational value of physical simulators as complementary tools in both undergraduate and postgraduate veterinary training. Simulation-based education facilitates repetitive practice, promotes progressive skill acquisition, and aligns with ethical imperatives such as the 3Rs principle by reducing reliance on animal models.

The findings of this review indicate a predominance of simulators focused on the acquisition of basic laparoscopic skills and a limited range of surgical procedures, primarily sterilization. Most simulators are low-fidelity and centered on a restricted number of species, predominantly canine. Although these models play a key role in early skills development, their limited scope constrains their applicability to real-world veterinary surgical practice. A significant lack of standardized and methodologically robust validation strategies was identified, thereby limiting the proper verification and comparative assessment of the proposed solutions. Validation processes were highly heterogeneous and were frequently conducted by participants with limited experience, increasing the risk of bias.

Accordingly, future research should prioritize the development of simulators addressing higher-complexity procedures, underrepresented species, and the implementation of rigorous and standardized validation methodologies. Strengthening the methodological quality and translational relevance of simulation research will be essential to maximize its impact on veterinary education, clinical performance, and animal welfare.

## Data Availability

The raw data supporting the conclusions of this article will be made available by the authors, without undue reservation.
